# Electroglottography in the diagnosis of functional dysphonia

**DOI:** 10.1007/s00405-018-5012-6

**Published:** 2018-07-30

**Authors:** A. Szkiełkowska, P. Krasnodębska, B. Miaśkiewicz, H. Skarżyński

**Affiliations:** 1 Audiology and Phoniatrics Clinic, Institute of Physiology and Pathology of Hearing, Warsaw, Poland; 2 Audiology and Phoniatrics Faculty, Fryderyk Chopin University of Music, Warsaw, Poland; 3 Otorhinolaryngology Clinic, Institute of Physiology and Pathology of Hearing, Warsaw, Poland

**Keywords:** Electroglottography, Dysphonia, Vocal fold, Glottography, Articulation

## Abstract

**Introduction:**

Electroglottography (EGG) is the most commonly used method of indirect visual examination of vocal fold vibration.

**Aim:**

The study was conducted with an aim of assessing EGG quasi open quotient (QOQ_EGG_) in different functional dysphonias to develop a differential diagnosis. The second aim was to check the influence of articulation on QOQ_EGG_ values.

**Material and methods:**

There were 20 people without voice problems, 20 patients with hypofunctional dysphonia and 20 patients with hyperfunctional dysphonia included in the study. Electroglottography was recorded during comfortable sustained phonation of [a], [e], [i], [o], [u].

**Results:**

There were no statistically significant differences in QOQ_EGG_ observed during phonation of different vowels in the control group and patients with hyperfunctional dysphonia. In patients with hypofunctional dysphonia, significantly higher values of QOQ_EGG_ were observed during [a] and [e]. Both in the control and in studied groups vowel [i] was vocalized significantly quieter.

**Conclusions:**

To conclude, EGG can be useful in differential diagnosis of functional dysphonia. QOQ_EGG_ is a parameter differentiating hypofunctional dysphonia from hyperfunctional dysphonia. Dissimilarities in articulation of different vowels in patients with various types of dysphonia influence values of QOQ_EGG_. EGG study protocol in cases of functional dysphonia should include a comparison of [a], [e], [i] vowels.

## Introduction

Electroglottography (EGG) is the most commonly used method of indirect visual examination of vocal fold vibration. The method was first described by Fabre in 1957 as high frequency glottography [1].

EGG measures electric impedance between two electrodes placing them against the skin, overlying each thyroid lamina. Between the electrodes flows the current of low-voltage and low-amperage. Its impedance changes with movements of vocal folds [2]. Weak high frequency electrical signal that is generated form the EGG electrode does not produce any sensation and does not result in tissue damage, muscle contraction, or nerve stimulation [3]. Main advantages of this method are non-invasiveness and lack of influence on the process of articulation and voice creation. In 1958, Timcke et al. assigned phases of the glottal cycle to the EGG curve [4]. The authors compared the position of vocal folds visualized by a high speed video recording with the shape of electroglottogram and on the basis of those observations defined the open quotient OQ_EGG_ as the duration of the opening phase divided by the time of whole glottal cycle.

Through subsequent dozens of years, researchers questioned the usefulness of OQ_EGG_ [5]. This was mainly due to difficulties related to unambiguous determination of the opening of vocal folds. The researchers agreed that any clinical conclusion based on an electroglottogram would be highly subjective [5]. Introduction of a quasi-open quotient QOQ_EGG_ in the beginning of XXI century restored the practical value of EGG. Observations of the parameter in functional disorders published by Jilek and co-authors encourage the use of QOQ_EGG_ measures [6]. What is more, changes in the EGG recording, such as bifurcations which occur in cases of vocal fold pathology [7, 8] do not seem to influence automatic calculations of QOQ_EGG_.

### Aim

The study was conducted with an aim of determining the utility of QOQ_EGG_ in differential diagnosis of two different forms of functional dysphonia. The second aim was to check the influence of articulation on QOQ_EGG_ values.

### Materials and methods

Only women were included in this study due to the disproportionate representation of women in functional dysphonia. The control group included healthy volunteers, who subjectively assessed their voice as normal. All subjects underwent auditory-perceptual assessment of voice and laryngovideostroboscopic examination (LVS) by the authors [9]. There were 20 euphonic women without vocal complains included in the control group. The second group comprised women suffering from muscle tension dysphonia. Patients with organic lesions found in LVS were excluded from the study. 20 patients with hypofunctional dysphonia and 20 patients with hyperfunctional dysphonia were included. The diagnosis of muscle tension dysphonia was based on medical history, results of otolaryngologic and phoniatric examination and acoustic analysis [10]. Subjects were aged between 20 and 60 years.

**Fig. 1:**
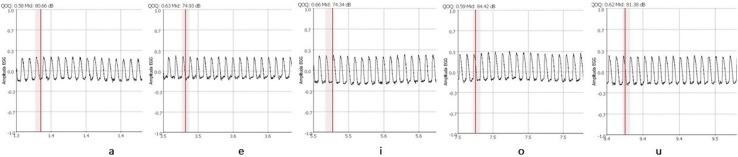
EGG waves during various vowels phonation in patient with hyperfunctional dysphonia

Electroglottography was recorded during comfortable sustained phonation of [a], [e], [i], [o], [u] (Fig. 1). According to Titze classification there are three types of EGG curve [7]. The first type presents periodic, regular recording. The second type is a record of periodic waves with bifurcations or subharmonics. The third and last type relates to a chaotic record without any regularity.

Electroglottography module of EndoSTROB Xion device allowed calculating the average sound pressure level in decibel (SPL dB) and QOQ_EGG_. QOQ_EGG_ is a parameter describing the relative open phase duration of a glottal cycle [11]. Each EGG wave is divided by a baseline in such a manner that the areas under and above the line are equal. Positive EGG wave (positive deflection of increasing impedance) is treated like an open phase. The ratio of the positive curve time to the duration of a whole cycle is defined as a quasi open quotient. The value of QOQ_EGG_ and the value of the sound pressure level in dB were calculated separately for each cycle.

In this study was analysed the middle and the most regular-shaped recording of sustained vowel phonation. The distance from MIC to mouth was 15 cm. The part of the recording was manually selected by one of the authors and then automatically analysed. The QOQ_EGG_ values and the SPL values were averaged from a minimum of 20 consecutive cycles [12].

The study design was approved by the Institutional Bioethics Committee.

Statistical analysis was performed using Microsoft Excel and the R statistical tests. Normality of all distributions was tested, and based on non-normal distributions, non-parametric statistical tests were used. The following tests were used:

- Mann–Whitney test, to examine the relationship between: values of QOQ_EGG_ and occurrence or type of dysphonia, values of parameter and vowel type, value of SPL dB generated by the subjects during phonation and vowel type.
- Significance test, Pearson correlation and Spearman correlation—to examine the relationship between: values of QOQ_EGG_ and age; values of parameter and the value of SPL dB generated by the subjects during phonation.

The level of statistical significance was set at *p* < 0.05.

## Results

The mean age in the control group was 47 years (SD = 12) and in the study group it was 42.5 years (standard deviation SD = 8.5) in patients with hyperfunctional dysphonia and 51.5 years (SD = 7) in patients with hypofunctional dysphonia. The average sound pressure level varied from 70 to 85 dB.

Mean values of QOQ_EGG_ differed between studied groups. In the control group, we observed differences of average values of SPL dB during phonation of vowels between the examined individuals, but these differences were not significant (Table 1). Different levels of voice intensity during comfortable phonation were also observed in patients with dysphonia (Table 2). Both in the control and in studied groups vowel [i] was vocalized significantly quieter (*p* < 0.05). There was no relationship between the values of QOQ_EGG_ and the value of the intensity of sound generated by the subjects during comfortable phonation.

**Table 1:** Mean values (with standard deviation) and medians (with minimal and maximal value) of QOQ_EGG_ and mean values (with standard deviation) of dB SPL of control group

	a			e			i			o			u		
	Mean value	Median	dB SPL	Mean value	Median	dB SPL	Mean value	Median	dB SPL	Mean value	Median	dB SPL	Mean value	Median	dB SPL
Control group	0.53	0.52	78.5	0.56	0.56	77.4	0.548	0.54	70	0.555	0.56	78	0.54	0.56	77
	0.054	0.44	3.68	0.045	0.45	3.5	0.054	0.405	5	0.06	0.42	4	0.058	0.45	3.6
		0.64			0.65			0.66			0.67			0.67	

**Table 2:** Mean values (with standard deviation) and medians (with minimal and maximal value) of QOQ_EGG_ and mean values (with standard deviation) of dB SPL of patients with functional dysphonia

	a			e			i			o			u		
	Mean value	Median	dB SPL	Mean value	Median	dB SPL	Mean value	Median	dB SPL	Mean value	Median	dB SPL	Mean value	Median	dB SPL
Hyperfunctional dysphonia	0.53	0.55	80	0.57	0.57	77	0.56	0.56	71.4	0.556	0.55	80	0.567	0.56	76
	0.053	0.41	3.44	0.039	0.44	3.7	0.04	0.49	3.5	0.04	0.44	4.26	0.05	0.49	8.26
		0.69			0.62			0.64			0.67			0.71	
Hypofunctional dysphonia	0.6	0.58	76	0.6	0.61	75	0.54	0.57	70.2	0.545	0.55	79	0.57	0.58	76
	0.055	0.48	6.54	0.02	0.5	4.5	0.04	0.41	4.55	0.06	0.41	6	0.05	0.47	5.7
		0.63			0.69			0.6			0.63			0.67	

**Fig. 2:**
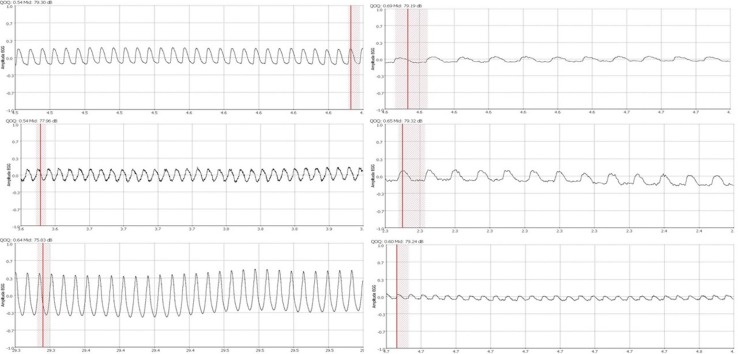
Various shapes of EGG waves of patients with hypofunctional dysphonia

All electroglottograms recorded in the study were readable; none has been classified as third grade according to Titze classification. In five people the recorded wave was slightly irregular (second degree according to Titze classification). In one of those cases the computer software had no difficulties in determining the QOQ_EGG_ of each glottal cycle. In the remaining four, due to irregularities of the EGG-wave, computer software could not designate 2–4 out of 20 tested cycles. Electroglottograms of patients with hyperfunctional dysphonia are characterized by a rapid closing phase of the vocal folds. Most patients had a sawtooth wave (Fig. 1). The variety of shapes was bigger in patients with hypofunctional dysphonia (Fig. 2). In those patients a mild increase of vocal fold closure draws attention.

The average value and median of QOQ_EGG_ in the control group are shown in Table 1. There were no statistically significant differences in QOQ_EGG_ observed during phonation of different vowels.

We did not obtain statistically significant correlation between the value of QOQ_EGG_ and age neither in the control nor in the studied group.

No statistically significant differences between the control group and the hyperfunctional dysphonia group in the values of QOQ_EGG_ were found. We observed a tendency for higher QOQ_EGG_ values during phonation of [i] in the control group compared to the studied group (*p* = 0.11).

In patients with hypofunctional dysphonia, significantly higher values of QOQ_EGG_ were observed during [a] and [e] phonation compared to other vowels (*p* < 0.05). The relation was also observed in comparison to healthy controls (*p* < 0.05). These patients phonated [a] significantly quieter than patients with hyperfunctional dysphonia and control group. Level of dB SPL during phonation of [e] was smaller in patients with hypofunction of the vocal folds in comparison to the control group.

## Discussion

Through analysis of the QOQ_EGG_ in various types of functional dysphonias we aimed to develop a successful differential diagnosis.

It is thought that change of the articulation does not influence EGG. However, the articulation does affect the aerodynamic characteristics of the air flowing through glottis and so it may induce changes in the vibratory characteristics of vocal folds [13]. In this work, we have supported that the articulation does not influence glottal cycle measured with EGG in healthy subjects and patients with hyperfunctional dysphonia, but in patients with hypofunctional dysphonia the QOQ_EGG_ values are higher during phonation of [a] and [e]. Zagólski and co-authors analysed the results of EGG obtained from 16 healthy women and 8 healthy men [14]. They analysed EGG values such as Qx (contact quotient of the vocal folds) and the index of irregularity. EGG was recorded during comfortable phonation of [a], [o], [u], “Ala”, “Ola”, “Ula”, and the sentence ”dzisiaj jest ładna pogoda” (English: *the weather is nice today*). Zagólski did not find any differences in Qx during different language tests. Similar observation was made by Kelman [15]. He did not observe any differences in Qx in healthy subjects during phonation of [a], [e], [i], [o] and [u].

The relation between the type of voice disorder and QOQ_EGG_ value has been described by Hacki [11] and Childers et al. [16]. Hacki published observations of nearly 170 people with and without voice disorders during crescendo. The values of QOQ_EGG_ in crescendo (from 55 to 90 dB SPL) in subjects with normal voice ranged from 0.4 to 0.75. The author noted that with the increase of dB SPL the QOQ_EGG_ in patients with normal voice and hyperfunctional dysphonia decreases. Inverse relationship, however, had been observed in people with hypofunctional dysphonia.

We have found significant differences in QOQ_EGG_ during comfortable phonation of [a] and [e] between individuals with different types of functional dysphonia. Mean values of QOQ_EGG_ in patients with hyperfunctional dysphonia were smaller compared to patients with hypofunctional dysphonia. We have also observed higher values of QOQ_EGG_ during phonation of [i] in patients with hyperfunctional dysphonia, which can be explained with a widening of a glottal gap caused by the increase of vocal fold tension when voice frequency is higher. Extension of the opening phase relative to the closing phase with the increase of voice frequency had been previously described by Childers [17].

We have not found any relation between the level of phonation and the value of QOQ_EGG_ during comfortable phonation. Independent studies carried out by Howard, Heinrich and Mooshammer have showed that the OQ_EGG_ and CQ_EGG_ change with volume and pitch of phonation [18–20]. OQ_EGG_ increases with growing frequency and decreases with growing intensity of voice. Similar observations of QOQ_EGG_ were published by Hacki [16]. In line with the principles of the present study the authors have adopted a safe (according to previous publications) range of SPL dB [21]. Patients were not forced to produce a sound on a pre-established level, but could adopt their individual level of comfortable phonation. Despite the differences in the level of comfortable phonation we did not obtain any statistically significant differences of the analysed parameters with relation to age.

According to our observations, the most severe problem during the examinations was a shortened time of phonation. We have encountered this condition more often than any irregularities during VLS although they are supposed to be the main limitation of VLS. Patients with dysphonia phonated for a shorter time and often several attempts of comfortable phonation were required to visualise the entire length of the glottis. Similar observations have been published by Hill et al. [22]. They compared acoustic, stroboscopic and electroglottographic evaluation of phonation. They stated that these methods are effective in evaluation of all patients without voice disorders, but only for 42% of patients with dysphonia. The authors had problems in the evaluation of 23% of VLS examinations, 46% of EGG and 35% of acoustic recordings. In most cases the difficulties in assessing were caused by patient’s inability to sustain long, stable phonation or by too irregular signal, primarily in patients with aphonia.

In the material of our work, which consisted of patients with functional dysphonias, we were able to analyse all VLS recordings. We have also obtained readable EGG waves in all studied cases. The material did not include patients with aphonia.

## Conclusions

Electroglottography can be useful in differential diagnosis of functional dysphonia.

Quasi open quotient is a parameter differentiating hypofunctional dysphonia from hyperfunctional dysphonia.

Electroglottography methodology is crucial in cases of functional dysphonia because of dissimilarities in articulation of different vowels in patients with various types of dysphonia.

Comparison of vowels [a], [e], [i] should be included in the EGG study protocol.
